# Parvalbumin interneurons in the hippocampal formation of individuals with Alzheimer’s disease: a neuropathological study of abnormal phosphorylated tau in neurons

**DOI:** 10.3389/fnana.2025.1571514

**Published:** 2025-04-10

**Authors:** Paula Merino-Serrais, Sergio Plaza-Alonso, Silvia Tapia-Gonzalez, Gonzalo León-Espinosa, Javier DeFelipe

**Affiliations:** ^1^Laboratorio Cajal de Circuitos Corticales, Centro de Tecnología Biomédica, Universidad Politécnica de Madrid, Madrid, Spain; ^2^Departamento de Neurobiología Funcional y de Sistemas, Instituto Cajal, CSIC, Madrid, Spain; ^3^Centro de Investigación Biomédica en Red sobre Enfermedades Neurodegenerativas (CIBERNED), ISCIII, Madrid, Spain; ^4^Laboratorio de Neurofisiología Celular, Facultad de Medicina, Universidad San Pablo-CEU, CEU Universities, Madrid, Spain; ^5^Departamento de Química y Bioquímica, Facultad de Farmacia, Universidad San Pablo-CEU, CEU Universities, Urbanización Montepríncipe, Madrid, Spain

**Keywords:** interneurons, Alzheimer’s disease, tau phosphorylation, GABAergic paravalbumin, hippocampus

## Abstract

Alzheimer’s disease (AD) is the most common neurodegenerative disorder in the elderly. Recent efforts have centered on understanding early events that trigger AD, aiming to facilitate early diagnosis and intervention for improved patient outcomes. The traditional histopathological features observed in AD encompass the extracellular accumulation of amyloid-beta protein and the intracellular abnormal phosphorylation of Tau protein (pTau). However, elucidating how these pathological hallmarks ultimately contribute to cognitive deficits remains a complex challenge. While AD is commonly conceptualized as a disorder characterized by synaptic failure, substantial knowledge gaps persist regarding the mechanisms underlying the onset and progression of the disease, underscoring the need for novel and more effective therapeutic approaches. In this context, the impairment of GABAergic paravalbumin (PV+) neurons has been proposed as a crucial factor contributing to neuronal network dysfunction and cognitive decline in AD. The presence of pTau in pyramidal neurons is directly linked to their impairment in AD; however, the effect of pTau in PV+ neurons remains unclear. In this present study, we analyzed the existence of PV+ neurons containing pTau using immunocytochemistry in the hippocampal formation and entorhinal cortex of human samples from diagnosed AD cases and individuals without neurological or psychiatric disorders. Two pTau isoforms, pTau_AT8_ and pTau_pS396_, corresponding to early and late stages of AD respectively, were examined. Our findings indicate that most PV+ neurons across the hippocampal formation and entorhinal cortex did not contain pTau in either group cases. Interestingly, while AD cases diagnosed with dementia exhibited a higher number of pTau+ neurons, the majority of PV+/pTau+ neurons were found in individuals with no neurological alterations. This suggests that the presence of pTau in PV+ neurons does not directly correlate with the overall abundance of pTau+ neurons. Given that PV+ neuron impairment is a key pathogenic mechanism in AD and is associated with cognitive decline, understanding the changes in PV+ neurons during AD progression could provide critical insights into the alterations of neuronal circuits underlying the disease.

## Introduction

Numerous histopathological studies have focused on understanding the early events that trigger Alzheimer’s disease (AD), with the goal of facilitating early diagnosis and intervention to improve patient outcomes. Classical histopathological findings in the brains of individuals with Alzheimer’s disease include the accumulation of amyloid-beta (Aβ) protein in the neuropil, as well as abnormal phosphorylation (pTau) and aggregation of tau protein within neurons, leading to the formation of paired helical filaments of hyperphosphorylated tau protein, known as neurofibrillary tangles (Achúcarro, 1910).

Beyond Aβ plaques and NFTs, additional neuropathological changes, such as neuronal and synaptic loss, have been observed in AD (Raskin et al., 2015), along with the presence of intracellular Aβ (Friedrich et al., 2010). Among these, synaptic loss appears to be the most significant structural correlate of cognitive decline and is considered one of the earliest pathological mechanisms, preceding neuronal loss (Selkoe, 2002; Coleman et al., 2004; Arendt, 2009). The precise mechanisms by which these processes lead to cognitive deficits in AD remain unclear. It is widely believed that synaptic dysfunction, possibly due to the toxic effects of Aβ and pTau, plays a significant role in cognitive impairment associated with AD (e.g., Griffiths and Grant, 2023). However, numerous studies have shown that individuals without cognitive symptoms can accumulate Aβ plaques in their brains, and that neurofibrillary tangles may also be present in non-demented individuals, possibly as part of normal aging [for a recent review see de Vries et al. (2024)]. This raises two critical questions regarding AD pathology: to what extent do synaptic alterations explain early cognitive decline in AD, and how can these changes be ameliorated or prevented? It has been suggested that the connection between neuronal structural pathology in AD and clinical symptoms lies in an imbalance between excitatory and inhibitory circuits, which may be a key factor uniting the phenomena observed at various levels of analysis in AD (Canter et al., 2016; Frere and Slutsky, 2018; Giovannetti and Fuhrmann, 2019; Mondragón-Rodríguez et al., 2019; Mondragón-Rodríguez et al., 2020; Maestú et al., 2021; Benussi et al., 2022).

In this context, GABAergic interneurons expressing parvalbumin (for simplicity, PV+ neurons) have been the focus of significant interest due to their role in regulating network oscillations and maintaining the balance between excitation and inhibition (Canter et al., 2016; Frere and Slutsky, 2018; Giovannetti and Fuhrmann, 2019; Mondragón-Rodríguez et al., 2019; Mondragón-Rodríguez et al., 2020; Maestú et al., 2021; Benussi et al., 2022). As key orchestrators of gamma oscillations, PV+ neurons are thought to play a crucial role in synchronizing neuronal activity, shaping the encoding, consolidation, and retrieval of memories (Verret et al., 2012; Pelkey et al., 2015; Topolnik and Tamboli, 2022).

Several studies have reported that impairment of PV+ neurons is a key factor contributing to neuronal network dysfunction and cognitive decline in mouse models of AD (reviewed in Hijazi et al., 2023). Indeed, PV+ neurons are now being explored as a novel therapeutic target for AD (Wei et al., 2023).

The medial temporal lobe (MTL) is the first region affected in AD progresses (Braak and Braak, 1991; for recent studies, see, for example, Kobro-Flatmoen et al., 2021; Olajide et al., 2021; Zhang et al., 2024, and references therein). MTL circuits play a crucial role in declarative and spatial memory processes (Eichenbaum et al., 2012; Buzsáki, 2013; Berron et al., 2020) and encompass key brain areas, including the hippocampus; subiculum; parahippocampal and perirhinal cortices; and the entorhinal cortex (EC) (Mai and Paxinos, 2011). Of these, the CA1 field of the hippocampus, the subiculum and the EC are more severely affected in AD compared to other MTL areas (Hyman et al., 1984; Price et al., 1991; Morrison and Hof, 2002). Notably, significantly fewer PV+ neurons have been reported in the hippocampus, EC and perirhinal cortex of AD patients (Arai et al., 1987; Solodkin et al., 1996; Brady and Mufson, 1997; Mikkonen et al., 1999; Sanchez-Mejias et al., 2020).

However, it remains unclear whether PV+ neuron impairment in AD is directly related to the presence of pTau within these neurons, which typically occurs in pyramidal neurons and, in the case of the entorhinal cortex, also in stellate cells of layer II, or if it is due to other factors. Indeed, in one study using dual immunofluorescence to detect pTau with the antibody AT8 in PV+ neurons from AD individuals, it was found that the vast majority of PV-immunoreactive cells did not contain pTau (Blazquez-Llorca et al., 2010). However, phosphorylation patterns have been shown to vary with disease progression (Luna-Muñoz et al., 2007; Virginia et al., 2009; Neddens et al., 2018; Regalado-Reyes et al., 2019; Barthélemy et al., 2020) and tau protein has over 80 potential phosphorylation sites (Hanger et al., 2009). Various antibodies recognizing distinct abnormal tau epitopes have been employed as immunocytochemical markers of pTau in the brains of individuals with AD. Thus, analyzing additional patients at different stages of the disease and using other pTau antibodies may provide further insights into this issue.

In the present study, we examined the presence of PV+ neurons containing pTau using double-label immunocytochemistry with antibodies against PV+ and two pTau isoforms: pTau_AT8_ and pTau_pS396_. We analyzed all fields of the human hippocampal formation and entorhinal cortex in AD cases diagnosed with dementia (late stage of the disease) as well as in individuals who have no known neurological or psychiatric alterations but show a relatively large number of neurons with NFT (individuals with medium-high Braak stages).

The AT8 antibody recognizes the epitope characterized by phosphorylation at Ser202/Thr205 (Goedert et al., 1995). This antibody is widely employed, particularly in classifying neurofibrillary degeneration into Braak stages (Braak and Braak, 1991; Braak et al., 2006; Braak et al., 1994). Additionally, labeling with AT8 antibody appears to be associated with degenerative changes of the neuronal cytoskeleton, particularly during the early stages of AD (Goedert et al., 1995; Su et al., 1994; Kimura et al., 1996; Braak et al., 1994). For the pTau_p396_ isoform, we used the pS396 antibody which recognizes the epitope phosphorylated at site Ser396, commonly linked to the later stages of AD (Moloney et al., 2021). Furthermore, a previous study of the human hippocampus in individuals with late-stage AD, employing double immunofluorescence techniques, revealed that 64% of labeled neurons expressed only pTau_pS396_, 28% displayed both markers, and 8% showed only pTau_AT8_-ir (Furcila et al., 2019). Therefore, we used both antibodies in this study, since they label different subpopulations of neurons containing pTau.

Our results indicate that, despite numerous neurons containing pTau_AT8_ and pTau_pS396_ in the cases examined, the vast majority of PV+ neurons throughout the hippocampal formation in medium-high Braak stage individuals with or without dementia do not express pTau_AT8_ or pTau_pS396_.

## Materials and methods

### Sampling procedure

Human brain tissue (*n* = 10) was obtained at autopsy from: the Instituto de Neuropatología (IF4, IF5, IF8, IF10, IF13; Servicio de Anatomía Patológica, IDIBELL-Hospital Universitario de Bellvitge, Barcelona, Spain), the Neurological Tissue Bank (BCN1, BCN2, BCN12; Biobanc-Hospital Clínic-IDIBAPS, University of Barcelona, Spain) and the Banco de Tejidos Fundación CIEN (VK18, VK28; Centro Alzheimer, Fundación Reina Sofía, Madrid, Spain). All cases were obtained following national laws and international ethical and technical guidelines on the use of human samples for biomedical research purposes. Cases IF4, IF5, IF8, IF10 and IF13 showed no evidence of neurological alterations or dementia (non-demented; ND), whereas cases BCN1, BCN2, BCN12, VK16 and VK28 were diagnosed as AD patients with dementia by the above-mentioned centers (Table 1). For simplicity, we will refer to these two groups of individuals as ND (non-demented) and ADD (Alzheimer’s disease with dementia) cases. Since the numbers of males and females in each group were not balanced (Table 1; Supplementary Information), sex was not taken into consideration in our study. Briefly, upon removal, the brain was immediately fixed in cold 4% paraformaldehyde in phosphate buffer (PB: 0.1 M, pH 7.4), after which small blocks of the different regions of the cerebral cortex were obtained (10x10x10 mm). Subsequently, the blocks were post-fixed in the same fixative solution for 24 h at 4°C. After fixation, coronal sections (50 μm) of the cortical tissue were obtained from each region with a vibratome and immunohistochemistry was then performed. The sections immediately adjacent to these sections were Nissl-stained in order to identify the cortical areas and the laminar boundaries. In all cases, the time between death and tissue processing was lower than 5.5 h (Table 1).

**Table 1:** Clinical and neuropathological information of the human cases.

Case	Sex	Age (years)	Postmortem delay (h)	Braak stage	CERAD score	Additional diagnosis	Neuropsychological diagnosis
IF4	F	82	3	III	B		ND
IF5	F	80	3	III	0	TAD	ND
IF8	M	91	3	III	A	AGD	ND
IF10	M	66	3	I	A		ND
IF13	M	75	2–2.5	II	B	AGD	ND
BCN1	M	80	4.5	VI	C		ADD
BCN2	F	70	2	V	C		ADD
BCN12	F	74	3.5	V	C	SVD	ADD
VK16	F	88	2	VI	C	HS	ADD
VK28	F	86	5.5	V	C	LBD; HS	ADD

M, male; F, female; TAD, tangle-predominant variant of Alzheimer’s disease; AGD, argyrophilic grain disease; LBD, Lewy body disease; HS, hippocampal sclerosis.

The neuropathology of the cases was classified according to Braak stages (I–VI) (Braak and Braak, 1991) and CERAD Scores (0–C) (Mirra et al., 1991). Braak stages range from the absence of neurofibrillary tangles (0), to tangles primarily confined to the transentorhinal region (stage I and II), with additional involvement of limbic regions, including the hippocampus (stage III and IV), and finally, extensive neocortical involvement (stages V and VI), ultimately affecting primary motor and sensory areas. The CERAD Scores range from the absence of neuritic plaques (0) to sparse (A), moderate (B), or frequent (C) neuritic plaques in cortical regions of the superior and middle temporal gyri, middle frontal gyrus, and inferior parietal lobule.

### Immunohistochemistry

Single and double immunohistochemistry were performed on coronal sections of the hippocampal formation and EC using the following antibodies: rabbit anti-PV (PV, 1:1000, AB11427: Abcam), guinea pig anti-PV (1:200, 195004: Synaptic systems), mouse anti-tau (clone AT8, 1:2000, MN1020: Thermo Scientific, RRID: AB_223647; recognizes tau protein doubly phosphorylated at Ser202 and Thr205), and rabbit anti-tau (clone pS396; 1:2000, 44752G, RRID: AB_2533745, Invitrogen Corp., Carlsbad, CA, United States; recognizes tau protein phosphorylated at serine 396 in the C-terminal region).

For single immunohistochemistry, free-floating sections were pre-treated with 1.66% H_2_O_2_ for 30 min to remove the endogenous peroxidase activity. Subsequently, the slices were blocked for 1 h in PB with 0.25% Triton-X and 3% normal goat serum (S-2000, Vector Laboratories). The sections were then incubated 48 h at 4°C with rabbit anti-PV antibody. The following day, the sections were rinsed and incubated for 2 h with biotinylated horse anti-rabbit IgG antibodies (1:200, BA-1000: Vector Laboratories). Antibody binding was detected using the Vectastain ABC immunoperoxidase kit (Vector Laboratories) and visualized with the chromogen 3,3′-diaminobenzidine tetrahydrochloride (DAB; Sigma-Aldrich). After staining, the sections were dehydrated, cleared with xylene, and covered-slipped using DePeX (100579, Merck KGaA). A BX51 Olympus microscope was used for all imaging.

For double immunohistochemistry, free-floating sections were blocked for 1 h in PB with 0.25% Triton-X and 3% normal goat serum. The sections were then incubated overnight at 4°C with the primary antibodies described above. The next day, the sections were incubated for 2 h at room temperature with a mixture of Alexa fluor-conjugated secondary antibodies: goat-anti mouse 594 and goat-anti rabbit 488 (1:1000, A11005 and A11008, respectively; Molecular Probes). Alternatively, sections were incubated with biotinylated goat anti-guinea pig secondary antibody (1:200; BA-7000; Vector Laboratories), followed by a mixture of Alexa fluor 594 anti-rabbit (1:1000, A11012, Molecular Probes) and streptavidin coupled to Alexa fluor 488 (1:1000; S32354; Molecular Probes). After rinsing in PB, the sections were treated with Autofluorescence Eliminator Reagent (2160, Chemicon) to reduce lipofucsin-like autofluorescence, as autofluorescence is a potential confounding factor (Stillman et al., 2023), due to the accumulation of lipofuscin with age (Terman and Brunk, 1998). The sections were washed and mounted with ProLong Gold Antifade Reagent (P36930, Thermofisher).

To confirm the specificity of the primary antibodies for DAB and fluorescence immunostaining, negative controls were conducted in parallel with the primary experiments, either by omitting the primary antibody under the same conditions or by using inappropriate secondary antibodies to assess non-specific binding and background signal. Under these conditions, no labeling was observed (Supplementary Figure 1).

### Confocal imaging acquisition and quantification

Imaging was performed with a ZEN inverted scanning confocal system (Zeiss LSM 710; Carl Zeiss Microscopy GmbH, Jena, Germany). Fluorescence signals from Alexa 488 (green) and Alexa 594 (red) were captured through separate channels. Confocal images encompassing the entire hippocampus were acquired with a 10x objective lens (NA, 0.3), and composite panoramic images were constructed using ZEN 2012 software (Zeiss). Higher amplification images were acquired using a 20x objective lens (NA, 0.8).

After image acquisition, one panoramic image from each case was manually analyzed using Neurolucida 360 software (MicroBrightField Inc., Williston, VT). The areas of interest were first traced, including the pyramidal layer of the hippocampus proper, divided into CA1 (CA1-Pyr), CA2 (CA2-Pyr), CA3 (CA3-Pyr) and CA4 (CA4-Pyr), as well as the granule cell layer and polymorphic layer (or hilus) of the dentate gyrus (DG). CA4 field refers to the Ammon’s horn neurons located within the concavity of the granule cell layer of the DG. The stratum oriens was also traced for CA1 (CA1-Or), CA2 (CA2-Or) and CA3 (CA3-Pyr). Additional regions analyzed included the subiculum (Sub) and the entorhinal cortex (EC), which was divided into superficial layers (ECx-Sup; Layers II-III) and deep layers (ECx-d; Layers V-VI). Tracings of the hippocampal regions were based on 50 μm-thick immunostained sections. The red channel was then hidden, and all visible PV+ neurons were marked within the selected areas. To determine whether the PV+ neurons also expressed pTau_AT8_ or pTau_pS396_, the red channel was reactivated, and each PV+ neuron was individually examined. Additionally, to quantify the number of pTau_AT8_ and pTau_pS396_ labeled neurons in each area of interest, the green channel was hidden, and all pTau_AT8_ or pTau_pS396_ positive neurons were marked.

Finally, the analyzed areas were exported to Neurolucida Explorer (MicroBrightField Inc., Williston, VT) for quantitative analysis. The results are presented as the number of PV+, pTau_AT8_ or pTau_pS396_ positive neurons per mm^2^ of area in the different regions studied (Tables 2, 3; Supplementary Tables 1, 2).

**Table 2:** Total number of PV+ neurons individually analyzed in the hippocampal formation and entorhinal cortex.

	CA1-Pyr	CA2-Pyr	CA3-Pyr	CA4-Pyr	CA1-Or	CA2-Or	CA3-Or	Sub	ECx-Sup	ECx-d
ND	234 (1)	57 (1)	14	37	58	14	12	811 (4)	693	193
ADD	108	30 (1*)	16	14	91 (1)	11	6	278 (1*)	412 (1)	61
Total	342	87	30	51	149	25	18	1,089	1,105	254

Numbers in brackets indicate the total number of PV+ neurons expressing pTau_AT8_ or pTau_pS396_ (denoted with an asterisk).

**Table 3:** Total number of pTau neurons individually analyzed in the hippocampal formation and entorhinal cortex.

	CA1-Pyr	CA2-Pyr	CA3-Pyr	CA4-Pyr	CA1-Or	CA2-Or	CA3-Or	Sub	ECx-Sup	ECx-d
ND	397 (1)	53	5	8	8	3	1	140 (2)	56	36 (2)
ADD	3,179 (2,028)	112 (59)	119 (35)	143 (57)	232 (218)	73 (69)	8	1,244 (1,043)	465 (223)	114 (48)
Total	3,576	165	124	151	240	76	9	1,384	521	150

Numbers in brackets represent the total number of neurons expressing pTau_pS396_, while the remaining numbers represent those expressing pTau_AT8_.

## Results

**Figure 1:**
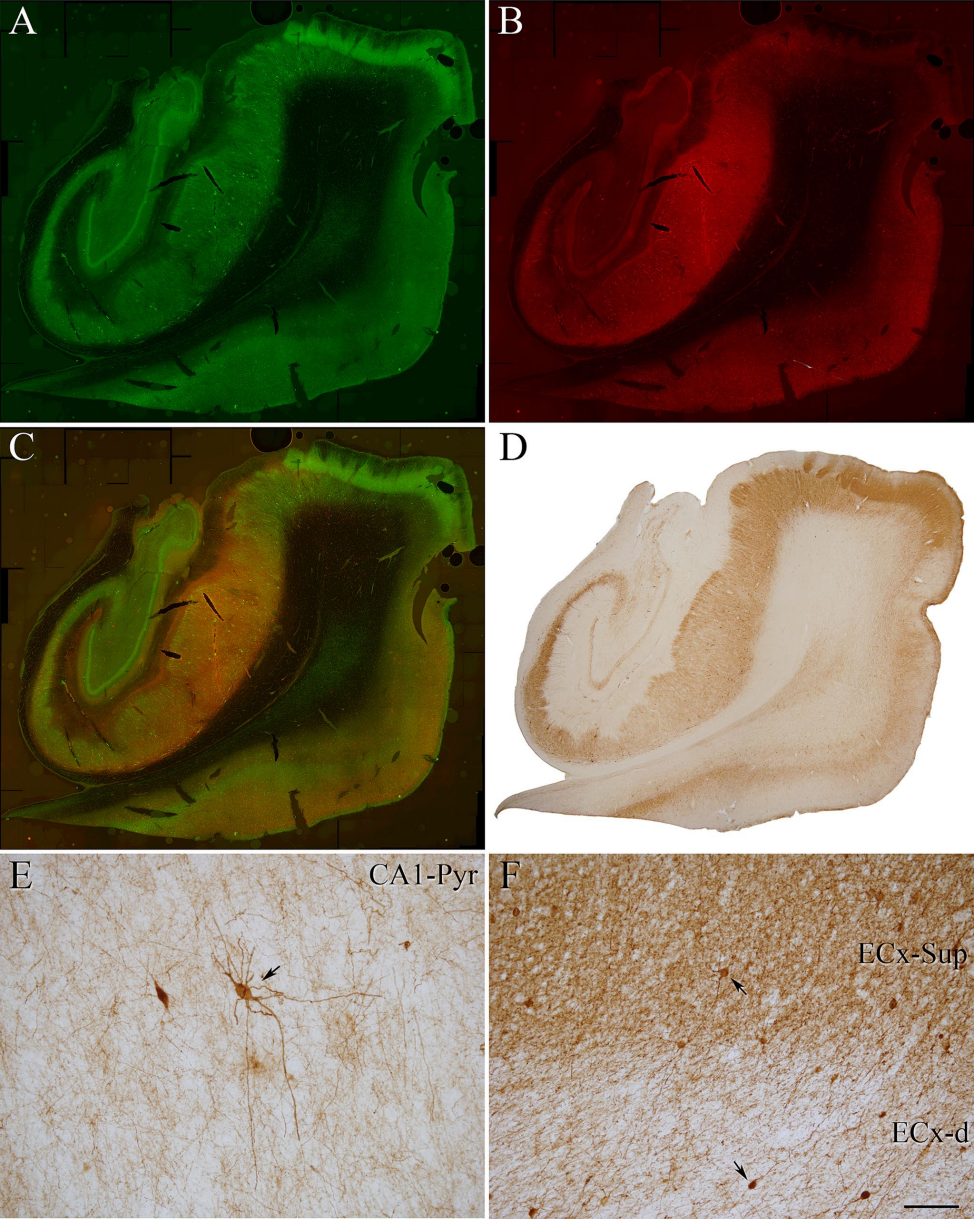
Coronal sections of human hippocampal formation from an ND case (IF4). **(A,B)** Panoramic confocal images double stained for PV (green) **(A)** and pTau_AT8_ (red) **(B)**. **(C)** Composite image obtained by combining **A,B**. **(D–F)** Photomicrograph showing the distribution of PV+ neurons in a DAB-immunostained section adjacent to the sections shown in **A,B**. **(E,F)** Higher magnification of **D** showing PV+ neurons (indicated with arrows) in the CA1 pyramidal layer **(E)** and in the EC **(F)**. Scale bar shown in **F** indicates 1,200 μm in **A–D** and 30 μm in **E,F**.

**Figure 2:**
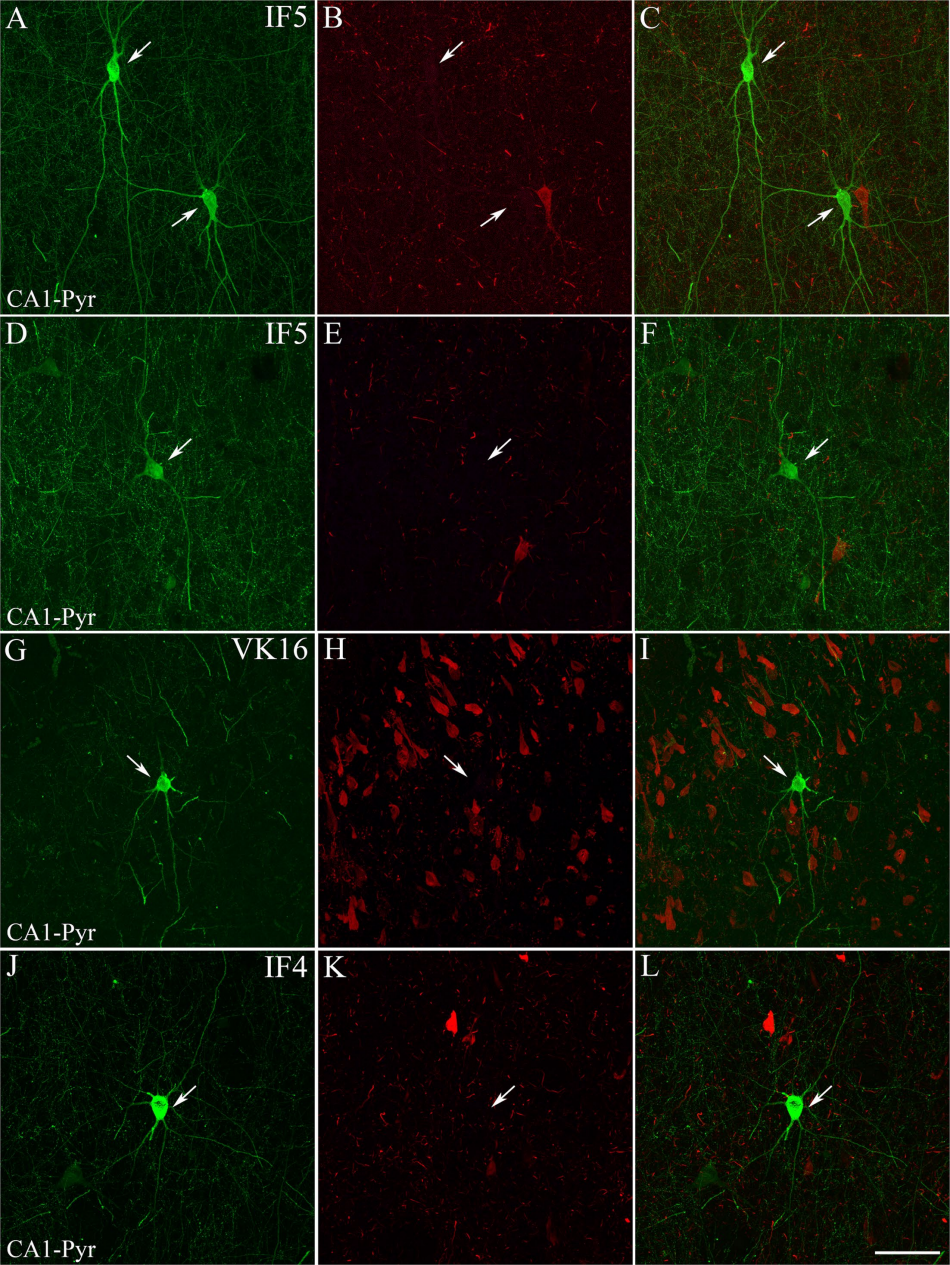
Examples of hippocampal PV+ neurons that are not labeled for pTau. Confocal images of double-immunostained sections for PV (green; **A,D,G,J**) and pTau_AT8_ (red; **B,E,H,K**). **(C,F,I,L)** Composite images obtained by combining the corresponding left panels. PV+ neurons (arrows) are not stained for pTau_AT8_. Images are from the CA1 pyramidal layer from ND cases IF5 **(A–F)** and IF4 **(J–L)** — and ADD case VK16 **(G–I)**. Scale bar shown in L indicates 30 μm for all panels.

**Figure 3:**
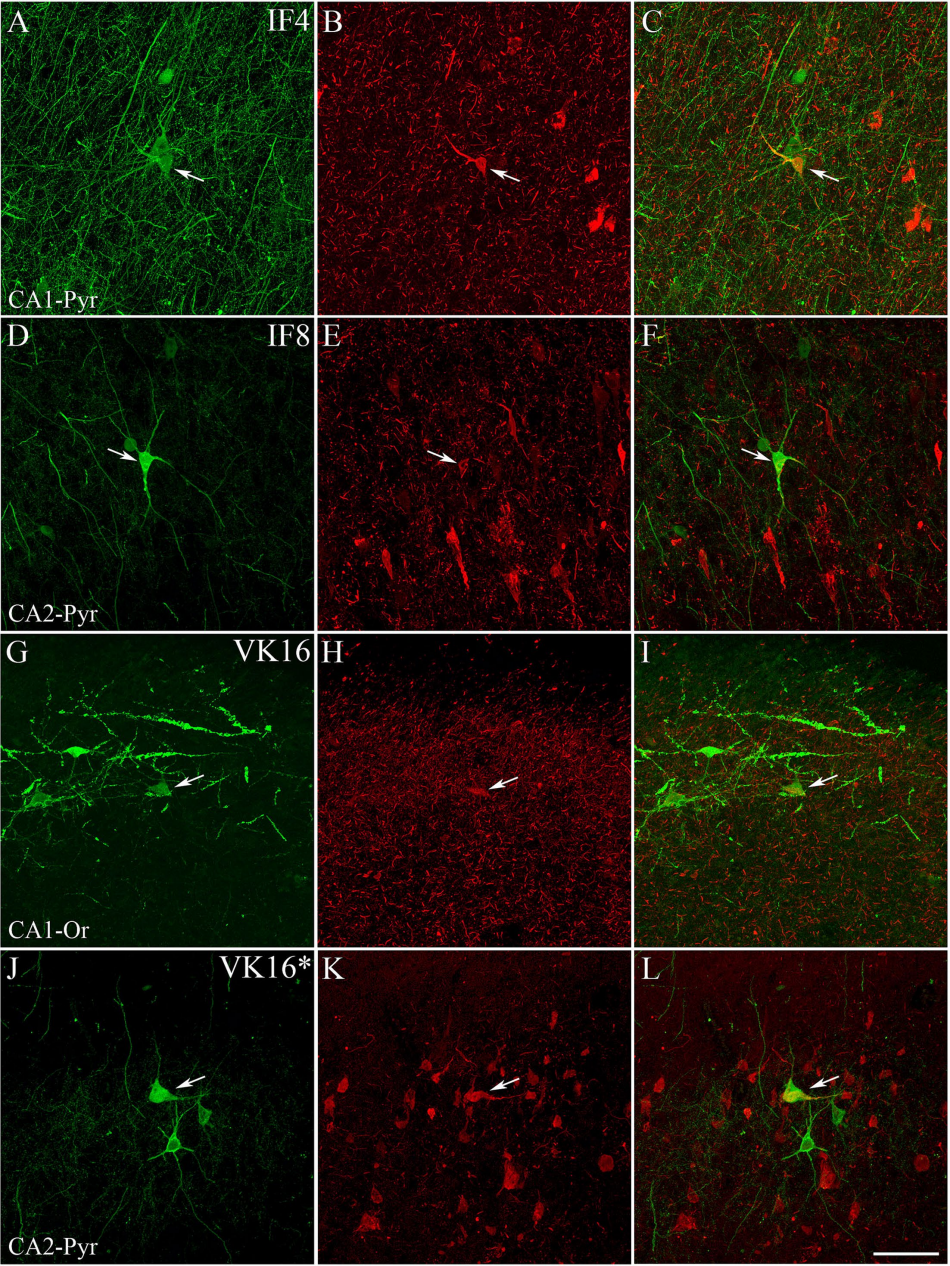
Examples of hippocampal PV+ neurons also labeled for pTau. Confocal images of double-immunostained sections for PV (green; **A,D,G,J**) and pTau_AT8_ (red; **B,E,H**) or pTau_pS396_ (red; **K**). **(C,F,I,L)** Composite images obtained by combining the corresponding left panels. Some PV+ neurons (arrows) are stained for pTau_AT8_ or pTau_pS396_. Images were taken from the CA1 pyramidal layer from ND cases IF4 **(A–C)**, IF8 **(D–F)** and ADD VK16 **(G–L)**. See Supplementary Figure 2 for higher magnification images of these panels. Scale bar shown in L represents 30 μm for all panels.

**Figure 4:**
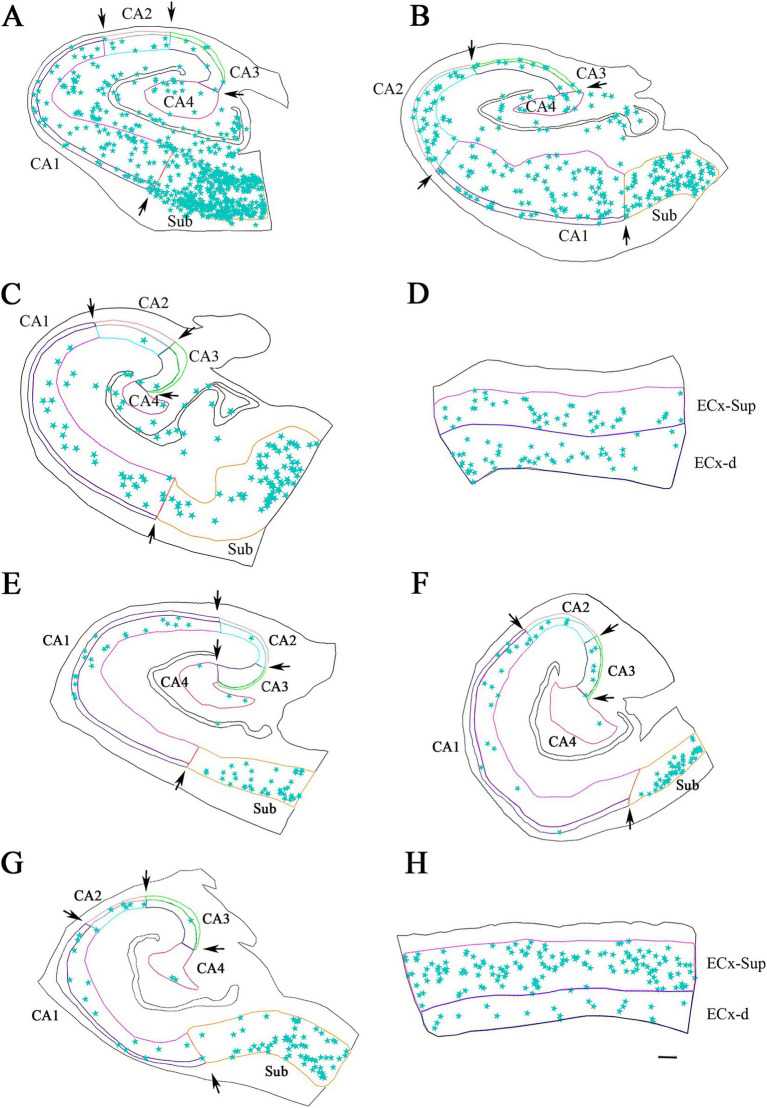
Neurolucida plot drawings showing the distribution of PV+ neurons in the hippocampal formation from ND and ADD cases. PV+ neurons are indicated with blue stars. Borders between the different cytoarchitectonic regions are indicated by arrows, whereas the colored lines mark the pyramidal cell layer in the hippocampus and granular cell layer of the DG, as well as the superficial and deep layers of the ECx. Areas studied from ND cases: IF8 **(A)**, IF4 **(B,D)** and IF5 **(C)**; and ADD cases: BCN1 **(E)**, VK28 **(F)**, BCN2 **(G)** and VK16 **(H)**. CA1, CA2, CA3, CA4 (Cornu Ammonis CA fields); ECx-Sup, superficial layers (II-III) of the entorhinal cortex; ECx-d, deep layers (V-VI) of the entorhinal cortex; Sub, subiculum. Scale bar: 600 μm in **A–C,F–H** and 350 μm in **D,H**.

We conducted a double-immunocytochemical analysis on coronal sections (Figures 1–3; Supplementary Figures 2, 3) from human cases (*n* = 10) with varying degrees of pTau and Aβ pathology and differing cognitive impairment diagnoses (see Table 1). In these coronal sections, each of the PV+ (Figure 4) and pTau+ neuron were plotted (Supplementary Figures 4, 5), generating a distribution map of the labeled neurons in the hippocampus and EC. Our analysis encompassed different regions from the hippocampal formation to assess potential differences based on specific anatomical characteristics within these fields. We focused on the pyramidal layer of the CA fields of the hippocampus (CA1-Pyr, CA2-Pyr, CA3-Pyr, and CA4-Pyr), as well as the stratum oriens (CA1-Or, CA2-Or and CA3-Or). The pyramidal and polymorphic layers of the Sub and the granule cell layer of the DG were also examined. However, as shown in Figure 4, PV+ neurons were virtually absent in the DG of ADD cases and most ND cases; consequently, the DG was excluded from the quantitative analysis.

In addition, we analyzed both the superficial (II-III) and deep layers (V-VI) of the entorhinal cortex (ECx-Sup and ECx-d, respectively). The stratum radiatum of the hippocampus and layer I of the entorhinal cortex were excluded from PV+ neuron counts due to the infrequent presence of PV+ neurons in these layers (see Andrioli et al., 2007).

A total of 2,123 PV+ neurons were counted in the hippocampal formation in ND cases, encompassing both the hippocampus and the EC, across a total analyzed surface of 147.6 mm^2^. In ADD cases, 1,027 PV+ neurons were counted within the same regions, over a total analyzed area of 107.03 mm^2^ (see Table 2; Supplementary Table 1). In total, 3,150 PV+ neurons were counted across both ND and ADD cases. The mean density of PV+ neurons per mm^2^ was similar between ND and ADD cases (mean ± SD: 11.2 PV+ ± 15.3 neurons/mm^2^ and 9.7 PV+ ± 15.8 neurons/mm^2^, respectively; see Supplementary Table 1 for detailed regional and individual data). However, given that atrophy is typical in the medial temporal lobe in AD, it is likely that the actual numbers of PV+ are higher in ND cases compared to ADD cases. In both ND and ADD cases, most PV+ neurons were found in the subiculum and the upper layers (II/III) of the EC (Table 2).

**Figure 5:**
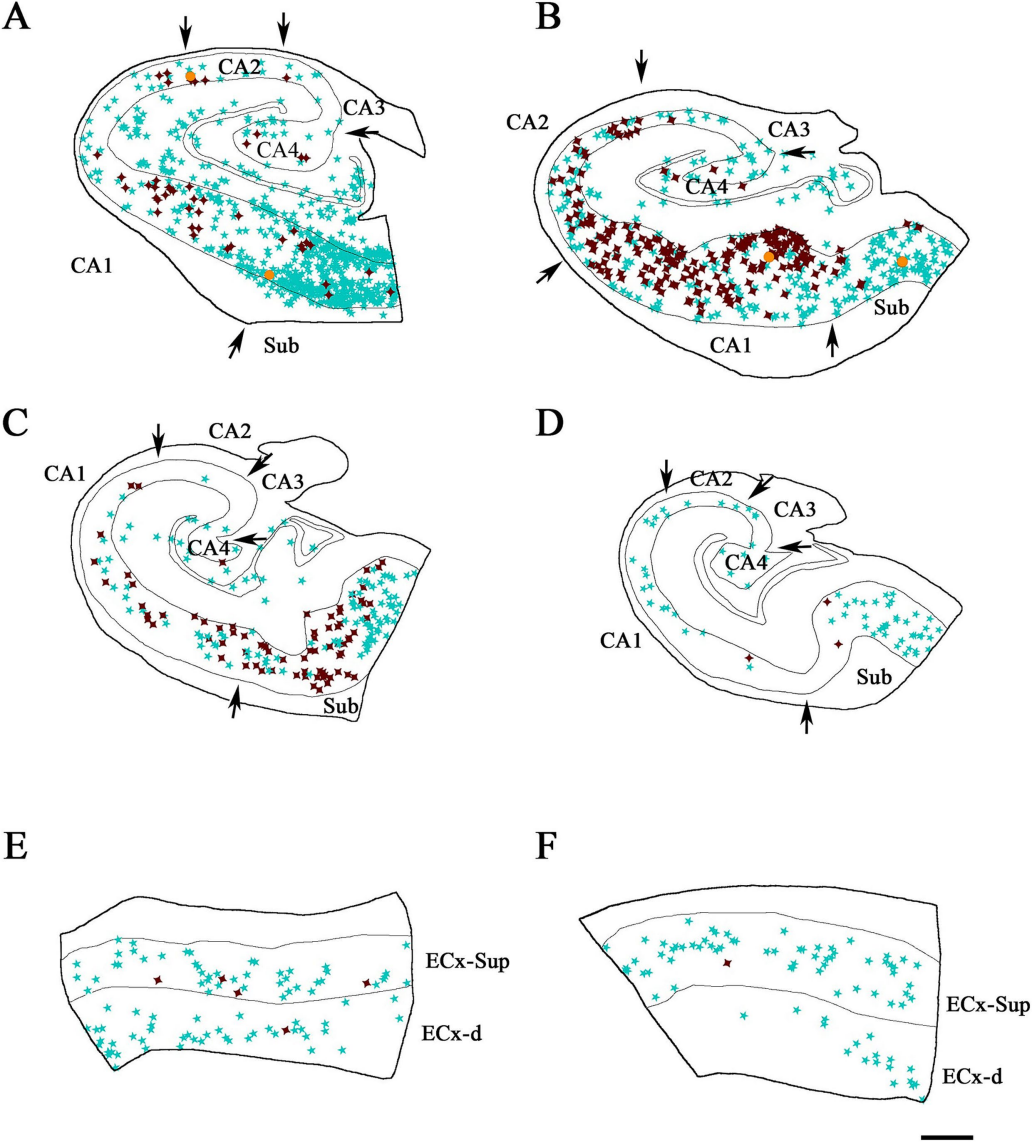
Illustrative drawings showing the distribution of PV+ and pTau neurons in the hippocampal formation and entorhinal cortex from ND cases. PV+ neurons are indicated with blue stars, pTau neuron are represented in brown, and PV+/ pTau_AT8_ **(A–C,E,F)** or PV+/pTau_pS396_ **(D)** neurons are indicated with orange dots. The borders between the different cytoarchitectonic regions are marked by arrows, and the thin continuous lines within each plot represent the pyramidal cell layer in the hippocampal formation and the granular cell layer of the dentate gyrus. See Supplementary Figure 4 for the Neurolucida plots illustrating the distribution of pTau-positive neurons only in ND cases. Areas studied from cases: IF8 **(A)**, IF4 **(B,E)** and IF5 **(C,D,F)**. CA1, CA2, CA3, CA4 (Cornu Ammonis fields); Sub (subiculum); ECx-Sup and ECx-d, superficial (II-III) and deep (V-VI) layers, respectively, of the entorhinal cortex **(E,F)**. Scale bar in F: 800 μm in **A–D** and 500 μm in **E,F**.

**Figure 6:**
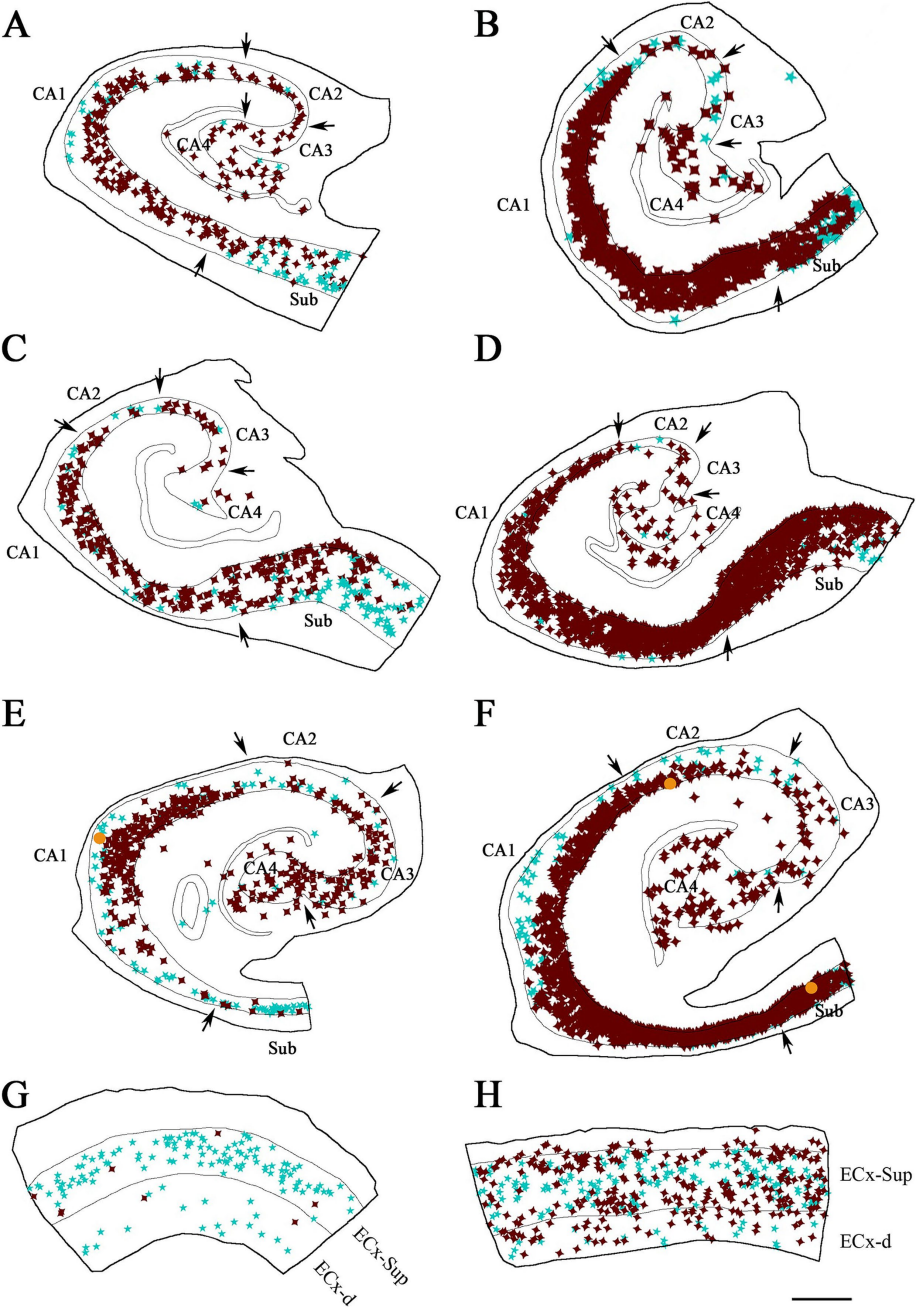
Illustrative drawings showing the distribution of PV+ and pTau+ neurons in the hippocampal formation and entorhinal cortex from ADD cases. PV+ neurons are indicated with blue stars, pTau neuron are represented in brown, and PV+/pTau_AT8_ **(A–C,E,G,H)** or PV+/pTau_pS396_ **(D,F)** neurons are indicated with orange dots. The borders between the different cytoarchitectonic regions are marked by arrows, and the thin continuous lines within each plot represent the pyramidal cell layer in the hippocampal formation and the granular cell layer of the dentate gyrus. The specific case analyzed is labeled in each drawing. See Supplementary Figure 5 for the Neurolucida plots illustrating the distribution of pTau-positive neurons only in ADD cases. Areas studied from cases: BCN1 **(A)**, VK28 **(B)**, BCN2 **(C,D,G)** and VK16 **(E,F,H)**. CA1, CA2, CA3, CA4 (Cornu Ammonis fields); Sub (subiculum); ECx-Sup and ECx-d, superficial (II-III) and deep (V-VI) layers, respectively, of the entorhinal cortex (G, H). Scale bar in H: 800 μm in **A–F** and 600 μm in **G,H**.

To determine whether PV+ neurons express pTau_AT8_ in the hippocampal formation, we analyzed PV+ neurons that were also labeled for pTau_AT8_ in double-labeled sections (PV+/pTau_AT8_) (Figures 5, 6). Out of the 2,746 PV+ neurons identified in sections immunostained with pTau_AT8_ (Supplementary Table 1), only 8 PV+ neurons exhibited pTau_AT8_ staining in their soma, representing 0.3% of the total cells analyzed (Table 2; Figures 5, 6; Supplementary Figures 4, 5).

A total of 6,396 pTau-positive neurons were counted across different regions of the hippocampal formation (Table 3; Supplementary Table 2). Of these, 2,611 neurons were labeled with pTau_AT8_, and 3,785 neurons were positive for pTau_pS396_. Specifically, ND cases presented 707 pTau-positive neurons (702 pTau_AT8_+ and 5 pTau_pS396_+; total analyzed surface: 147.60 mm^2^), whereas, ADD cases showed 5,689 pTau-positive neurons (1909 pTau_AT8_+ and 3,780 pTau_pS396_+; total analyzed surface: 107.03 mm^2^). In ND cases, the mean densities of pTau-positive neurons were 3.3 ± 5.8 pTau_AT8_+ neurons/mm^2^ and 0.1 ± 0.3 pTau_pS396_+ neurons/mm^2^. In ADD cases, higher densities were observed: 14.2 ± 18.2 pTau_AT8_+ neurons/mm^2^ and 76.9 ± 83.7 pTau_pS396_+ neurons/mm^2^ (see Supplementary Table 2 for details by region and individual). In both ND and ADD cases, most pTau-positive neurons were located in the CA1 and the subiculum (see Table 3).

As shown in Figure 3 and Supplementary Figure 2, PV+ neurons that were also immunostained for pTau exhibited labeling for pTau not only in their soma, but also in their proximal dendrites.

In addition, to investigate whether the proportion of PV+ neurons labeled for pTau differed depending on the pTau antibodies used, three additional cases (BCN2, IF5, and VK16) were analyzed with the pTau_pS396_ antibody. Only five pTau_pS396_ neurons were identified in ND cases, while 3,780 pTau_pS396_ neurons were observed in ADD cases. Similar to the results obtained with pTau_AT8_, double-labeled cells were rare: out of a total of 404 PV+ neurons examined in sections immunostained with pTau_pS396_, only two PV+ neurons also exhibited pTau_pS396_ (Figures 5, 6; Supplementary Table 1).

Overall, our findings indicate that the vast majority of PV+ neurons across the hippocampal formation did not contain pTau in either ND or ADD cases. Interestingly, although ADD cases exhibited a higher number of pTau+ neurons, the majority of PV+/pTau+ neurons were found in ND cases. This suggests that the presence of pTau in PV+ neurons is not related to the overall abundance of pTau+ neurons.

## Discussion

A number of studies implicate PV+ neuron dysfunction as one of the key pathogenic mechanisms in AD and the memory impairment associated with this disease (Wei et al., 2023). Consequently, understanding the earliest changes in PV+ neurons during AD progression could provide critical insights into the alterations of neuronal circuits underlying the disease. Studies of the hippocampal formation, entorhinal cortex and perirhinal regions reported a significant loss of PV+ neurons in AD patients (Arai et al., 1987; Satoh et al., 1991; Solodkin et al., 1996; Brady and Mufson, 1997; Mikkonen et al., 1999; Takahashi et al., 2010; Sanchez-Mejias et al., 2020). Since pTau is associated with severe neuronal loss, it is possible that the accumulation of pTau is toxic to PV+ neurons, leading to their degeneration and loss. However, our results demonstrate the virtual absence of pTau in PV+ neurons across different regions of the hippocampal formation and entorhinal cortex, despite pronounced tau pathology, regardless of dementia diagnosis. Indeed, only 10 out of 3,150 PV+ neurons displayed pTau. These findings corroborate our previous study showing the virtual absence of pTau in PV+ neurons of the medial temporal lobe in individuals with AD (Blazquez-Llorca et al., 2010). Therefore, although PV+ neuron loss has been proposed as another hallmark of AD, our data indicate that the mechanism underlying this loss is independent of intracellular pTau accumulation.

It is well-established from early neuropathological studies of AD patients, using silver staining methods, that in the cerebral cortex, pTau predominantly affect pyramidal neurons, ultimately leading to their destruction (Alzheimer’s Association, 2023; Alzheimer, 1907). Other neuron types have been reported to exhibit remarkable resistance to neurofibrillary lesions (Lewis et al., 1987; Braak et al., 1989; Hof et al., 1991; Morrison et al., 1998). This raises an important question about the fate of GABAergic interneurons that innervate pyramidal cells. Given that pyramidal neurons are highly susceptible to degeneration in AD, it remains unclear how the associated GABAergic interneurons, particularly PV+ neurons, are affected. These PV+ neurons include basket and chandelier cells (DeFelipe et al., 1989) that provide the main GABAergic innervation to the soma, proximal dendrites, and axon initial segments of pyramidal cells (Freund and Buzsáki, 1996; DeFelipe, 1997, 1999). In this regard, it is important to note that the distribution of axon terminals around the soma and proximal processes of pTau neurons does not seem to be altered, as it is indistinguishable from both control cases and from adjacent neurons that do not contain pTau (Blazquez-Llorca et al., 2010). Thus, basket and chandelier cells seem to be unaffected by tau pathologies, although alterations of synaptic connections may exist at the molecular or physiological level. Further studies will be necessary to resolve this question. The lower number of PV+ neurons reported in other studies within the cortical regions examined in the present study may represent a selective loss of PV+ neurons that were connected to pyramidal cells, which disappeared due to neurofibrillary lesions. The loss of these pyramidal cells, and consequently the postsynaptic targets of a subpopulation of PV+ neurons, could have induced the loss of the PV+ neurons that innervated them. The surviving PV+ neurons would continue to innervate the remaining pyramidal cells, regardless of whether these pyramidal cells contain pTau, or not.

The pathological pTau/interneuron association has been studied extensively in mouse models and, in general, it has been reported that most GABAergic interneurons are co-labeled with pTau. For example, in aged B6 mice injected with AD brain-derived extracellular vesicles, pTau_AT8_ labeling was found in PV+ and GAD67 GABAergic interneurons (Zhi et al., 2021). Additionally, in the 3xTg AD mouse model, pTau was detected in GABAergic interneurons labeled with GAD67, PV, and somatostatin (Mondragón-Rodríguez et al., 2020; Zheng et al., 2020). This contrasts with the results of both the present study and previous research on AD individuals, as discussed above. A major obstacle in interpreting data from mouse models in comparison to human cases is the inherent differences in brain structure and function, as well as the fact that not all aspects of the disease can be included in mouse models.

Detailed double-labeling experiments to study pTau in hippocampal GABAergic interneurons from AD cases are less frequent and difficult to compare with the present results. For example, Zheng et al. (2020) reported a significant accumulation of pTau in the subgranular cell zone and hilus of the dentate gyrus in both AD patients and mice, with the majority of these pTau-positive cells identified as GABAergic interneurons by co-labeling with GAD67, PV, and somatostatin. A major difference between Zheng et al.’s study and ours is the longer postmortem delay in their study and their use of an antigen-retrieval protocol for immunostaining, whereas we employed standard immunostaining, which included a treatment to reduce autofluorescence. The postmortem interval, brain tissue fixation, and immunostaining protocols may lead to significant changes in neuronal and glial integrity at the anatomical and neurochemical levels, as well as in metabolomic analyses (Gonzalez-Riano et al., 2017). Further studies using additional AD cases at different stages of the disease and other antibodies recognizing distinct abnormal tau epitopes are necessary to verify and extend the present results.

## Data Availability

The original contributions presented in the study are included in the article/Supplementary material, further inquiries can be directed to the corresponding author.
