# Supplementary material for: Parvalbumin interneurons in the hippocampal formation of individuals with Alzheimer’s disease: a neuropathological study of abnormal phosphorylated tau in neurons
Source: Front Neuroanat. 2025 Apr 10;19:1571514. doi: 10.3389/fnana.2025.1571514 (PMC12018435; doi:10.3389/fnana.2025.1571514)
Supplement: Supplementary file 1 [file Data_Sheet_1.pdf]

Parvalbumin Interneurons in the Hippocampal Formation of Individuals with Alzheimer's Disease: A Neuropathological Study of abnormal phosphorylated tau in neurons

Paula Merino-Serrais[1,2,3*], Sergio Plaza-Alonso[1,2,3], Silvia Tapia-Gonzalez[4], Gonzalo León-Espinosa[1,5], Javier DeFelipe[1,2,3]

[1]Laboratorio Cajal de Circuitos Corticales, Centro de Tecnología Biomédica, Universidad Politécnica de Madrid, Madrid, Spain

[2]Departamento de Neurobiología Funcional y de Sistemas, Instituto Cajal, CSIC, Madrid, Spain

[3]Centro de Investigación Biomédica en Red sobre Enfermedades Neurodegenerativas (CIBERNED), ISCIII, Madrid, Spain

[4]Laboratorio de Neurofisiología Celular, Facultad de Medicina, Universidad San Pablo-CEU, CEU Universities, Madrid, Spain

[5]Departamento de Química y Bioquímica, Facultad de Farmacia, Universidad San Pablo-CEU, CEU Universities, Urbanización Montepríncipe, 28660, Boadilla del Monte, Madrid, Spain

* Corresponding author:
Paula Merino-Serrais. Laboratorio Cajal de Circuitos Corticales, Centro de Tecnología Biomédica, Universidad Politécnica de Madrid, Campus Montegancedo S/N, Pozuelo de Alarcón, 28223 Madrid / Instituto Cajal (CSIC), Avenida Doctor Arce, 37, 28002 Madrid, Spain. Tel: (+34) 910679292 / paula.merino-serrais@cajal.csic.es
https://orcid.org/0000-0002-1842-9476

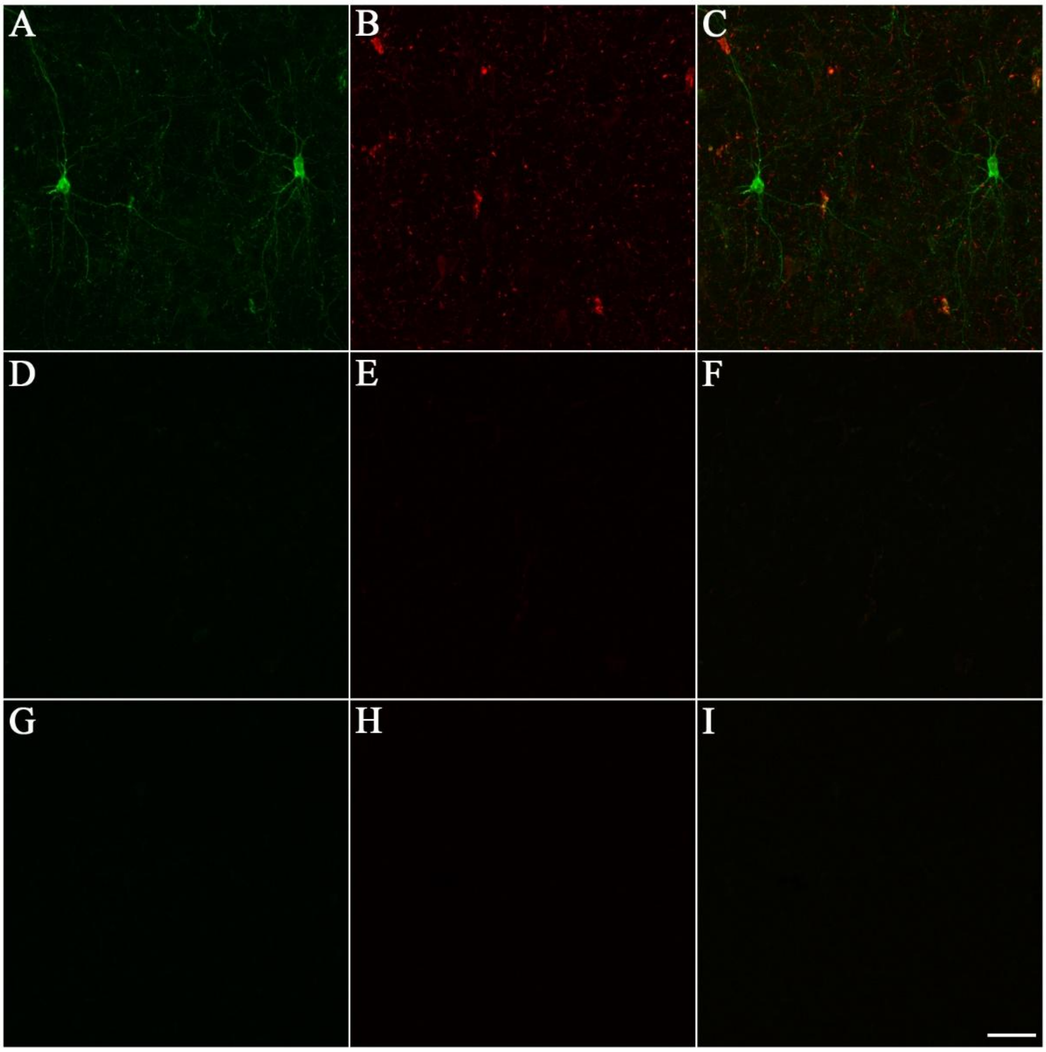

Supplementary Figure 1. Confocal images of double-immunostained sections for PV (green; A) and pTau $_{AT8}$ (red; B). (C) Composite image obtained by combining the corresponding left panels. Negative controls were conducted in parallel under the same conditions to the primary experiments and imaging acquisition, by omitting the primary antibody (D-F) or by using inappropriate secondary antibody (G-I; biotinylated goat anti-guinea pig secondary antibody, followed by streptavidin coupled to Alexa fluor 488). No labeling was observed under these conditions. These images were taken from CA1 pyramidal layer of an ADD case (95 years old male, Braak stage V and CERAD Score B, 4 hours of postmortem delay). Scale bar shown in I indicates 50 µm.

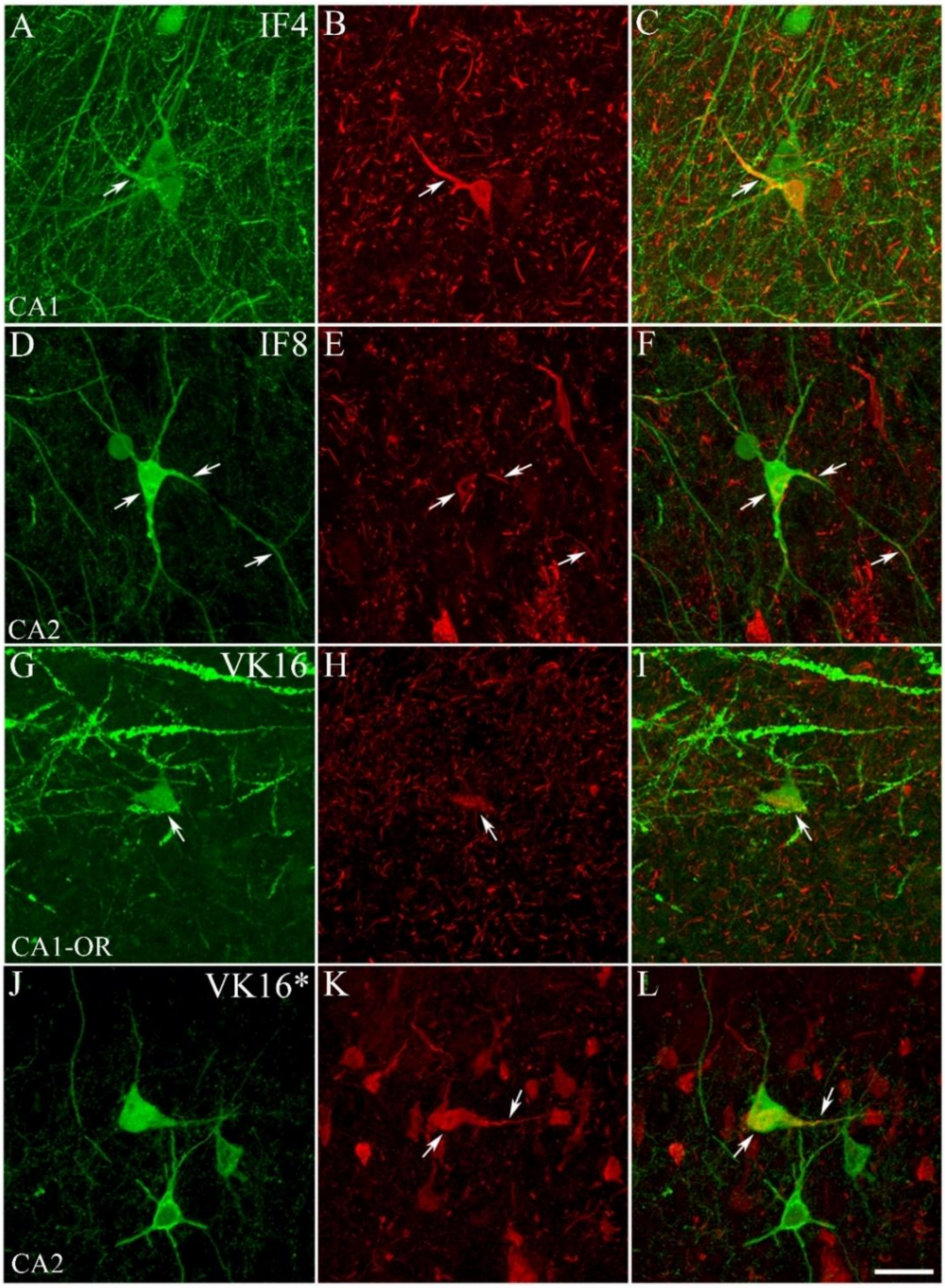

Supplementary Figure 2. Higher magnification of Figure 3 showing the presence of pTau in the soma and proximal dendrites in PV+ neurons. Confocal images showing PV+ neurons (green, A, D, G, J) and $pTau_{AT8}$ (red; indicated with an arrow; B, E, H) or $pTau_{pS396}$ (indicated with an arrow; K) in CA1 pyramidal layer from ND cases IF4 (A–C), IF8 (D–F) and ADD case VK16 (G–L). (C, F, I, L) Composite images obtained by combining the corresponding left panels. Scale bar shown in L indicates 15 μm.

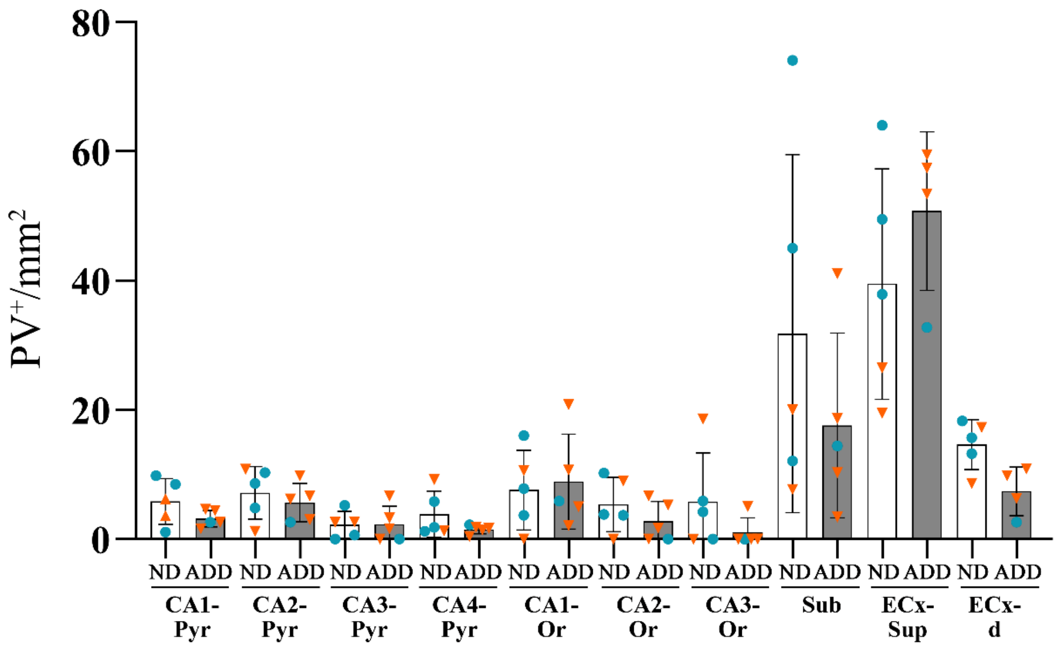

Supplementary Figure 3. Density of PV+ neurons in ND and ADD cases per region of interest. Values correspond to the number of PV+ neurons identified in each region divided by the total surface of the region. Each colored symbol represents a case (blue dots refers to male cases; orange triangles refer to female cases). Graph bars show mean values ± SD. Only sections labeled with $pTau_{AT8}$ were studied.

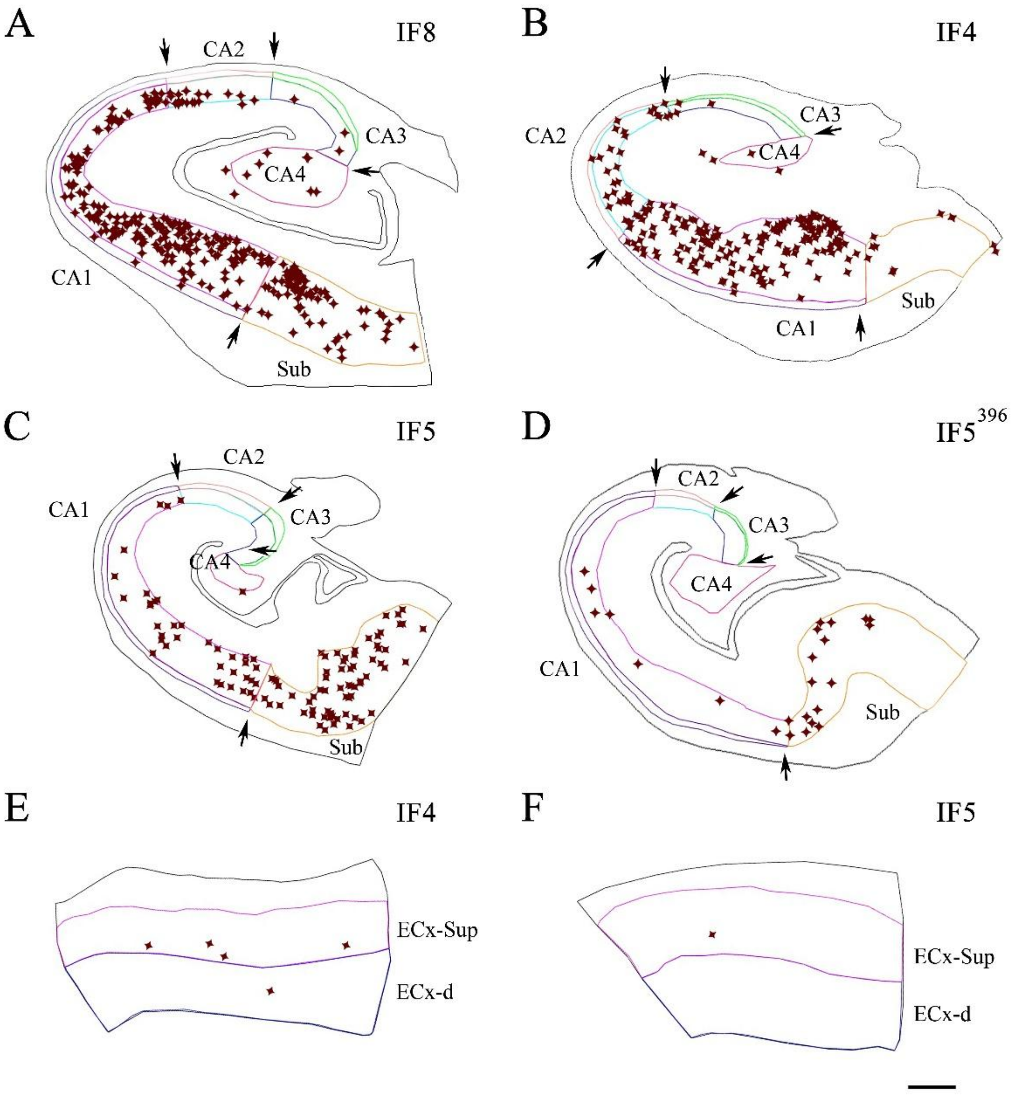

Supplementary Figure 4. Neurolucida plot drawings showing the distribution of pTau neurons in the hippocampal formation from ND cases. pTau neurons are indicated with brown stars for $pTau_{AT8}$ (A, B, C, E, F) and $pTau_{pS396}$ (D) staining. Borders between the different cytoarchitectonic regions are indicated by arrows, whereas the colored lines mark the pyramidal cell layer in the hippocampus and granular cell layer of DG, as well as the superficial and deep layers of the ECx. Abbreviations: CA1, CA2, CA3, CA4 (Cornu Ammonis CA fields); ECx-Sup, superficial layers (II-III) of the entorhinal cortex; ECx-d¸ deep layers (V-VI) of the entorhinal cortex; Sub, subiculum. CA4 field refers to the Ammon's horn neurons enclosed within the concavity of the

granule cell layer. Drawings taken from 50 µm-thick immunostained sections. Scale bar in F: 800 µm in A–D and 500 µm in E,F.

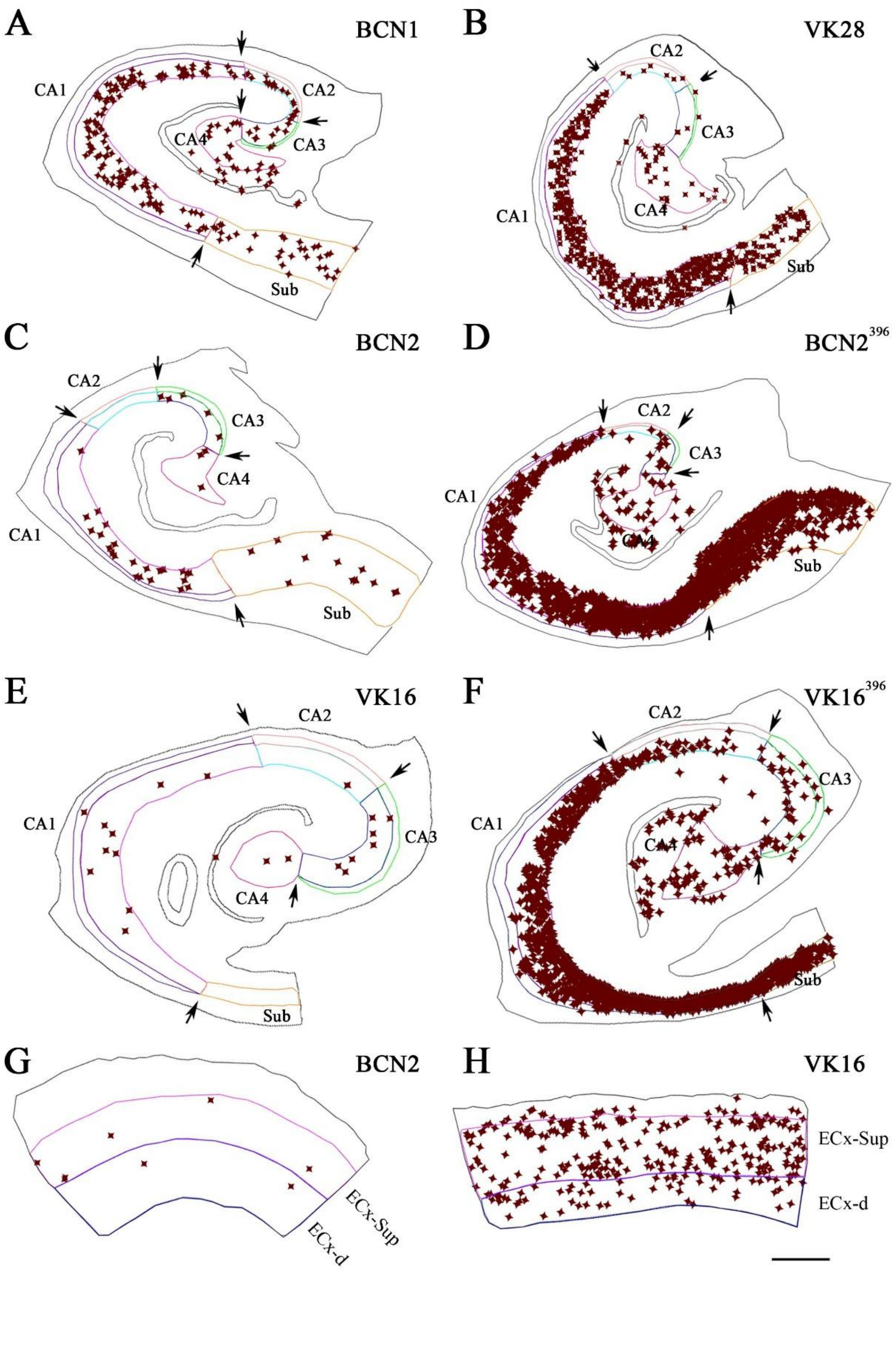

Supplementary Figure 5. Neurolucida plot drawings showing the distribution of pTau neurons in the hippocampal formation from ADD cases.

pTau neurons are indicated with brown stars for $pTau_{AT8}$ (A, B, C, E, G, H) and $pTau_{pS396}$ (D, F) staining. Borders between the different cytoarchitectonic regions are indicated by arrows, whereas the colored contour lines mark the pyramidal cell layer in the hippocampus and granular cell layer of DG, as well as the superficial and deep layers of the ECx, from cases BCN1, BCN2, VK16 and VK28. Borders between the different cytoarchitectonic regions are indicated by contours in the different hippocampal (A-F) and ECx (G, H) areas studied. Abbreviations: CA1, CA2, CA3, CA4 (Cornu Ammonis CA fields); ECx-Sup, superficial layers (II-III) of the entorhinal cortex; ECx-d¸ deep layers (V-VI) of the entorhinal cortex; Sub, subiculum. CA4 field refers to the Ammon's horn neurons enclosed within the concavity of the granule cell layer. Drawings taken from 50 µm-thick immunostained sections. Scale bar in H: 800 µm in A–F and 600 µm in G,H.

	Case		CA1-Pyr	CA2-Pyr	CA3-Pyr	CA4-Pyr	CA1-Or	CA2-Or	CA3-Or	Sub	ECx-Sup	ECx-d
**Non-Demented (ND)**	**IF4**	**N of PV+**	73 (1)	27	3	11	17	6	8	94 (1)	46	46
		**Area (mm^2)**	11.8	2.5	1.1	1.2	1.6	0.67	0.43	4.7	2.36	2.66
		**PV+/ mm^2**	6.19	10.80	2.73	9.17	10.63	8.96	18.60	20.00	19.49	17.29
	**IF5**	**N of PV+**	26	1	2	1	0	0	0	66	61	18
		**Area (mm^2)**	7.22	0.85	0.77	0.77	1.27	0.41	0.33	8.54	2.30	2.09
		**PV+/mm^2**	3.60	1.18	2.60	1.30	0	0	0	7.73	26.52	8.61
	**IF5***	**N of PV+**	19	2	3	5	0	0	0	39	78	5
		**Area (mm2)**	6.18	0.41	0.63	1.97	1.56	0.17	0.09	7.85	2.26	2.04
		**PV+/mm^2**	3.07	4.87	4.7	2.5	0	0	0	4.96	34.51	2.45
	**IF8**	**N of PV+**	60	9 (1)	5	13	23	1	2	357 (1)	258	34
		**Area (mm^2)**	6.15	1.05	0.96	2.26	1.44	0.26	0.34	4.82	4.03	2.58
		**PV+/μm^2**	9.76	8.57	5.21	5.75	15.97	3.85	5.88	74.07	64.02	13.18
	**IF10**	**N of PV+**	9	5	1	3	8	2	2	52	186	56
		**Area (mm^2)**	7.96	1.05	1.69	2.50	2.15	0.54	0.48	4.31	3.76	3.06
		**PV+/mm^2**	1.12	4.76	0.59	1.19	3.7	3.70	4.17	12.06	49.47	18.30
	**IF13**	**N of PV+**	47	13	0	4	10	5	0	203 (2)	64	34
		**Area (mm^2)**	5.53	1.26	0.34	2.17	1.29	0.49	0.079	4.51	1.69	2.17
		**PV+/mm^2**	8.48	10.31	0	1.84	7.72	10.20	0	45.01	37.87	15.67
**Alzheimer with dementia (ADD)**	**BCN 1**	**N of PV+**	12	1	0	3	8	0	0	42	67	4
		**Area (mm^2)**	4.67	0.38	0.61	1.36	1.36	0.19	0.090	2.91	2.04	1.52
		**PV+/mm^2**	2.56	2.63	0	2.20	5.84	0	0	14.43	32.84	2.63
	**BCN 2**	**N of PV+**	9	4	1	2	6	1	0	65	113	19
		**Area (mm^2)**	3.49	0.41	0.63	1.13	1.21	0.19	0.34	6.34	1.97	1.94
		**PV+/mm^2**	2.57	9.76	1.58	1.75	4.96	5.26	0	10.26	57.36	9.79
	**BCN 2***	**N of PV+**	5	1	0	2	2	1	0	30	-	-
		**Area (mm^2)**	5.04	0.24	0.39	1.35	1.30	0.09	0.07	5.06	-	-
		**PV+/mm^2**	0.99	4.16	0	1.48	1.54	11.11	0	5.90	-	-
	**BCN 12**	**N of PV+**	12	1	0	2	16	1	0	3	44 (1)	6
		**Area (mm^2)**	2.72	0.33	0.33	1.15	0.77	0.15	0.068	0.87	0.74	0.96
		**PV+/mm^2**	4.40	3.03	0	1.73	20.78	6.66	0	3.45	59.46	6.25
	**VK 16**	**N of PV+**	26	8	6	2	18 (1)	1	3	30	143	20
		**Area (mm^2)**	5.61	1.30	1.84	1.45	1.69	0.58	0.59	0.73	2.68	1.83
		**PV+/mm^2**	4.63	6.13	3.24	1.37	10.65	1.71	5.05	41.09	53.36	10.93
	**VK 16***	**N of PV+**	31	10 (1)	3	2	37	7	3	62 (1)	45	12
		**Area (mm^2)**	5.3	1.40	1.27	1.93	1.77	0.51	0.74	0.77	1.70	1.57
		**PV+/mm^2**	5.84	7.14	2.34	1.03	20.90	13.73	4.04	80.52	26.47	7.64
	**VK 28**	**N of PV+**	13	5	6	1	4	0	0	46	-	-
		**Area (mm^2)**	8.21	0.75	0.89	2.71	1.88	0.32	0.14	2.46	-	-
		**PV+/mm^2**	1.58	6.62	6.72	0.37	2.12	0	0	18.70	-	-

Supplementary Table 1. Total number of PV+ neurons analyzed per area of interest and case. Numbers in brackets represent the total number of PV+ neurons expressing pTau. * indicates p396 staining.

	Case		CA1-Pyr	CA2-Pyr	CA3-Pyr	CA4-Pyr	CA1-Or	CA2-Or	CA3-Or	Sub	ECx-Sup	ECx-d
**Non-Demented (ND)**	**IF4**	**N of AT8+**	163	23	2	1	4	3	1	7	4	1
		**Area (mm^2)**	11.8	2.5	1.1	1.2	1.6	0.67	0.43	4.7	2.36	2.66
		**AT8+/ mm^2**	13.81	9.2	1.81	0.83	2.5	4.47	2.32	1.48	1.69	0.37
	**IF5**	**N of AT8+**	23	0	0	0	0	0	0	52	1	0
		**Area (mm^2)**	7.22	0.85	0.77	0.77	1.27	0.41	0.33	8.54	2.30	2.09
		**AT8+/ mm^2**	3.18	0	0	0	0	0	0	6.08	0.43	0
	**IF5***	**N of 396+**	1	0	0	0	0	0	0	2	0	2
		**Area (mm^2)**	6.18	0.41	0.63	1.97	1.56	0.17	0.09	7.85	2.26	2.04
		**396+/ mm^2**	0.16	0	0	0	0	0	0	0.25	0	0.98
	**IF8**	**N of AT8+**	191	16	3	7	3	0	0	78	42	22
		**Area (mm^2)**	6.15	1.05	0.96	2.26	1.44	0.26	0.34	4.82	4.03	2.58
		**AT8+/ mm^2**	31.06	15.24	3.13	3.10	2.08	0	0	16.19	10.42	8.52
	**IF10**	**N of AT8+**	1	1	0	0	0	0	0	1	1	0
		**Area (mm^2)**	7.96	1.05	1.69	2.50	2.15	0.54	0.48	4.31	3.76	3.06
		**AT8+/ mm^2**	0.12	0.95	0	0	0	0	0	0.23	0.26	0
	**IF13**	**N of AT8+**	18	13	0	0	1	0	0	0	8	11
		**Area (mm^2)**	5.53	1.26	0.34	2.17	1.29	0.49	0.079	4.51	1.69	2.17
		**AT8+/ mm^2**	3.25	10.32	0	0	0.80	0	0	0	4.73	5.06
**Alzheimer with dementia (ADD)**	**BCN 1**	**N of AT8+**	194	19	9	18	0	0	2	32	25	5
		**Area (mm^2)**	4.67	0.38	0.61	1.36	1.36	0.19	0.090	2.91	2.04	1.52
		**AT8+/ mm^2**	41.54	50	14.75	13.23	0	0	22.22	10.99	12.25	3.28
	**BCN 2**	**N of AT8+**	177	2	15	4	4	0	0	72	6	2
		**Area (mm^2)**	3.49	0.41	0.63	1.13	1.21	0.19	0.34	6.34	1.97	1.94
		**PV+/mm^2**	50.71	4.87	23.80	3.54	3.31	0	0	11.36	3.04	1.03
	**BCN 2***	**N of 396+**	909	4	9	14	17	1	0	842	-	-
		**Area (mm^2)**	5.04	0.24	0.39	1.35	1.30	0.09	0.07	5.06	-	-
		**PV+/mm^2**	180.35	16.66	23.07	10.37	13.07	11.11	0	166.40	-	-
	**BCN 12**	**N of AT8+**	20	4	0	4	3	0	0	0	9	1
		**Area (mm^2)**	2.72	0.33	0.33	1.15	0.77	0.15	0.068	0.87	0.74	0.96
		**AT8+/ mm^2**	7.35	12.12	0	3.47	3.89	0	0	0	12.16	1.04
	**VK 16**	**N of AT8+**	211	22	57	35	3	3	6	6	202	58
		**Area (mm^2)**	5.61	1.30	1.84	1.45	1.69	0.58	0.59	0.73	2.68	1.83
		**AT8+/ mm^2**	37.61	16.92	30.97	24.13	1.77	5.17	10.16	8.21	75.37	31.69
	**VK 16***	**N of 396+**	1119	55	26	43	201	68	0	201	223	48
		**Area (mm^2)**	5.30	1.40	1.27	1.93	1.77	0.51	0.74	0.77	1.70	1.57

		**396+/ mm²**	211.13	39.28	20.47	22.27	113.55	133.33	0	261.00	131.17	30.57
	**VK 28**	**N of AT8+**	549	6	3	25	4	1	0	91	-	-
		**Area (mm²)**	8.21	0.75	0.89	2.71	1.88	0.32	0.14	2.46	-	-
		**AT8+/ mm²**	66.87	8.00	3.37	9.23	2.13	3.13	0	36.99	-	-

Supplementary Table 2. Total number of neurons expressing $pTau_{AT8}$ (AT8+) or $pTau_{pS396}$ (396+) per area of interest and case. * indicates p396 staining.
